# The Mental Imagery for Suicidality in Students Trial (MISST): study protocol for a feasibility randomised controlled trial of broad-minded affective coping (BMAC) plus risk assessment and signposting versus risk assessment and signposting alone

**DOI:** 10.1186/s40814-023-01273-7

**Published:** 2023-03-17

**Authors:** Peter James Taylor, Paula Duxbury, Jane Moorhouse, Chloe Russell, Dan Pratt, Sophie Parker, Chris Sutton, Fiona Lobban, Richard Drake, Steve Eccles, David Ryder, Rafeea Patel, Elizabeth Kimber, Eirian Kerry, Nathan Randles, James Kelly, Jasper Palmier-Claus

**Affiliations:** 1 Division of Psychology & Mental Health, University of Manchester, Manchester, UK; 2 Manchester Mental Health NHS Foundation Trust, Manchester, UK; 3 Division of Population Health, Health Services Research, and Primary Care, University of Manchester, Manchester, UK; 4 LA14YW, Spectrum Centre for Mental Health Research, Lancaster University, Lancaster, UK; 5 School of Medicine, Keele University, Newcastle-Under-Lyme, UK; 6 Doctorate in Clinical Psychology, Lancaster University, Lancaster, UK; 7 Lancashire & South Cumbria NHS Foundation Trust, Lancashire, UK

**Keywords:** Suicide, Self-harm, Students, Psychological therapy, Mental imagery

## Abstract

**Background:**

Going to university is an important milestone in many people’s lives. It can also be a time of significant challenge and stress. There are growing concerns about mental health amongst student populations including suicide risk. Student mental health and counselling services have the potential to prevent suicide, but evidence-based therapies are required that fit these service contexts. The Broad-Minded Affective Coping intervention (BMAC) is a brief (6 sessions), positive imagery-based intervention that aims to enhance students access to past positive experiences and associated emotions and cognitions. Pilot data provides preliminary support for the BMAC for students struggling with suicidal thoughts and behaviours, but this intervention has not yet been evaluated in the context of a randomised controlled trial (RCT). The Mental Imagery for Suicidality in Students Trial (MISST) is a feasibility RCT that aims to determine the acceptability and feasibility of evaluating the BMAC as an intervention for university students at risk of suicide within a larger efficacy trial. Key feasibility uncertainties have been identified relating to recruitment, retention, and missing data. Intervention acceptability and safety will also be evaluated.

**Method:**

MISST is a feasibility randomised controlled trial design, with 1:1 allocation to risk assessment and signposting plus BMAC or risk assessment and signposting alone. Participants will be university students who self-report experiences of suicidal ideation or behaviour in the past 3 months. Assessments take place at baseline, 8, 16, and 24 weeks. The target sample size is 66 participants. A subset of up to 20 participants will be invited to take part in semi-structured qualitative interviews to obtain further data concerning the acceptability of the intervention.

**Discussion:**

The BMAC intervention may provide an effective, brief talking therapy to help university students struggling with suicidal thoughts that could be readily implemented into university student counselling services. Depending on the results of MISST, the next step would be to undertake a larger-scale efficacy trial.

**Trial registration:**

The trial was preregistered (17 December 2021) on ISRCTN (ISRCTN13621293) and ClinicalTrials.gov (NCT05296538).

**Supplementary Information:**

The online version contains supplementary material available at 10.1186/s40814-023-01273-7.

## Introduction

Suicide is a leading cause of death amongst young people, both in the UK and globally [1, 2]. In the UK, there has been an increase in suicide rates amongst young people (e.g. those aged 10–24 years) in the last decade, which has also been mirrored by an increase in rates of self-harm amongst young people [3, 4]. Going to study at university is a key life event for many young adults, but there are also concerns about the mental health of students, including the risk of suicide [5, 6]. Whilst trends are difficult to identify due to the low base rate of the event, there is an indication that suicide rates may have increased amongst students in recent years, with recent estimated rates of 4.7 per 100,000 [7, 8]. Whilst these rates remain lower than for the non-student population, the suggested increase remains a concern. Moreover, university creates unique challenges for young people in accessing support (e.g. due to moving between termtime and home addresses), but also presents an opportunity for embedding interventions designed to prevent suicides that could reach a large population of young people during a key time in their lives [6].

Studying at university is characterised by major life transitions, academic stress, and financial pressures, which have been associated with suicide risk [5, 9, 10]. Research suggests that suicidal ideation is common amongst students, with one study indicating that 42% of students had contemplated suicide within a 12-month period [11]. Suicide attempt rates amongst UK students have been estimated at 4.9% (95% *CI*: 2.6–9.0%; [12]), although other studies suggest higher rates [13]. Suicidal ideation and attempts are predictors of future suicide risk and other forms of self-harm [14–16]. They are also indicators of psychological distress and mental health difficulties more generally [9, 11, 17]. Consequently, interventions aimed at supporting students experiencing recent suicidal ideation or behaviour may help reduce suicide risk and distress more generally. Moving away from home can represent a break from existing support systems, which may leave some individuals more vulnerable [18].

Demand on NHS mental health services is high and students may find themselves on long waiting lists for interventions or lost in the gap between NHS and university-based services [19]. This is problematic since the value of rapid access to psychosocial therapies for people struggling with suicidal thoughts or self-harm has been emphasised [20]. Students may face barriers to accessing and retaining continuity of input from NHS services when moving between home and termtime addresses and service providers. Most universities have dedicated student counselling or well-being services [6], which face increasing demand [21]. Working with students at risk of suicide can be a particular concern and challenge for university counselling staff [22]. Having effective interventions to help reduce suicide risk amongst students that can be readily implemented within university counselling service settings may not only be beneficial in the short term but may help foster longer-term resilience at a critical point in many young peoples’ lives.

There is growing evidence that a variety of psychosocial interventions may help to reduce suicidal thinking and self-harm, including cognitive behavioural therapy (CBT) and dialectical behaviour therapy (DBT; [23–25]). Many trials, however, focus on specific clinical settings (e.g. emergency departments) or diagnostic groups (e.g. borderline personality disorder), and do not consider university students as a distinct population. Whilst many therapies, such as CBT, work directly with negative or distressing cognitions and emotional states, another approach is to instead focus on enhancing access to positive emotions and cognitions.

The broad-minded affective coping (BMAC) technique [26, 27] uses mental imagery and savouring to strengthen a person’s access to and experience of positive memories, based on the theory that suicidal thinking may develop as part of a latent network of cognitions and emotions, with these connections becoming strengthened through repeated activation of these thoughts and feelings [28–30]. Thus, over time, a person’s suicidal thoughts may become more sensitive to activation and elaborated, increasing suicide risk [31, 32]. The BMAC provides a way of countering the activation and strengthening of this negative, suicide-related network, by instead reinforcing an incompatible network of positive thoughts, feelings, and memories [33, 34].

Online, self-guided administration of the BMAC was associated with short-term increases in positive affect amongst university students [36]. We developed the BMAC into a six-session therapy, with a focus on building clients’ capacity to apply the BMAC technique and access positive memories and emotions. A single-arm pilot study of this BMAC intervention with university students experiencing recent suicidal ideation or behaviour supported the feasibility and acceptability of this approach [34], with a decline in suicidal ideation after 6 and 12 weeks (*d* = 1.22–1.25). The BMAC therefore has promise a brief therapy for students experiencing recent suicidal ideation or behaviour.

The MISST study (Mental Imagery for Suicidality in Students Trial) is a feasibility randomised controlled trial (RCT) that aims to evaluate the feasibility, acceptability, and safety of the BMAC intervention for university students experiencing recent suicidal thoughts or behaviour. In order to ensure that risk is thoroughly and appropriately assessed and managed, both groups will also receive up to two sessions of risk assessment and signposting with a trained clinician, plus treatment as usual from student mental health services. Given that the focus of this trial is on the feasibility of evaluating the BMAC intervention in this context, there is no other active comparator. Feasibility has been operationalised in terms of the ability to recruit and retain participants and obtain outcome data on key measures, with minimal missing data. Acceptability has been operationalised in terms of adherence to therapy and qualitative feedback from participants regarding the therapy. Safety is being evaluated by monitoring adverse events and through qualitative feedback from participants. As a feasibility RCT, this study is not powered to evaluate treatment efficacy, but we will also exploratively examine change in key clinical outcomes (e.g. suicidal ideation, depression, hopelessness) to provide preliminary indications of the potential clinical promise of this approach and to inform parameters required for a larger-scale trial evaluation (e.g. sample size). Interviews with research and therapy staff will be used to further evaluate feasibility in undertaking a larger-scale efficacy-focused trial.

## Method

### Design

**Fig. 1:**
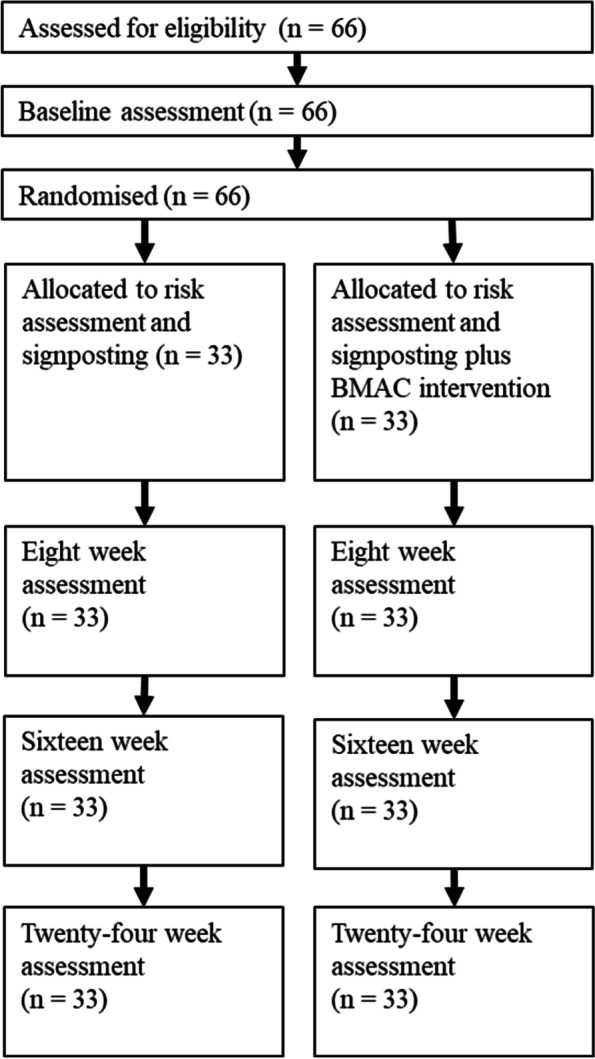
Trial flow diagram

This study will adopt an assessor blind feasibility RCT design with 1:1 allocation of participants to one of two trial arms. The two arms are (i) risk assessment and signposting (representing TAU) or (ii) risk assessment and signposting plus the BMAC intervention. Participants in both arms also have access to any concomitant treatments that form part of usual care, including interventions offered by university counselling services and those available through NHS. The trial includes assessment points at baseline and 8-, 16-, and 24-week post randomisation. This protocol follows the reporting guidelines set out in the Standard Protocol Items: Recommendations for Interventional Trials (SPIRIT) statement ([37]; see Supplementary Table 1). A flow chart for the trial procedures is presented in Fig. 1.

### Randomisation and blinding

Randomisation will be delivered via the independent online-sealed envelope service (sealedenvelope.com) using non-stratified, blocked randomisation (using computer-generated random permuted blocks or randomly varying block length). The principal investigators or trial manager will enter the participant ID into the website to obtain the allocation for that participant, which will then be communicated to the trial therapist. The study aims to randomise participants within three working days of completion of the baseline assessment. Researchers undertaking the follow-up assessments will be blind to participant allocation, and a research protocol will outline steps to avoid blind breaks (e.g. researchers not sharing an office with therapists, having non-masked trial documents stored in separate electronic folders that cannot be accessed by masked staff). Participants, referrers, and clinicians will be reminded regularly not to reveal group allocation to MISST research assistants. All blind breaks will be monitored. If a blind break occurs, then where possible, another researcher would complete the remaining assessments with that participant. Blinding can also be broken, when necessary, in response to immediate risk or medical emergencies. Trial statisticians will also be blinded until the statistical analysis plan is approved by the Trial Steering Committee (TSC) in order to avoid the plan being in any way influenced by an awareness of emerging data across the two trial aims.

### Participants

The population of interest is higher-education university students who self-report suicidal thoughts or behaviour within the past 3 months. Inclusion criteria are as follows: (i) aged ≥ 18 years; (ii) accessing full- or part-time education through a higher education institution (HEI); and (iii) suicidal ideation or behaviours in the past 3 months. The last criterion will be ascertained using the questions ‘Have you had any thoughts about ending your life in the past three months?’ and ‘Have you attempted to end your life in the past three months?’ Endorsement of either item will confirm eligibility for the trial and progression to full assessment. This approach is consistent with previous trials [38].

Exclusion criteria are as follows: (i) active/historical full-threshold first-episode psychosis or bipolar disorder as identified by the patient or referring service and the MINI diagnostic interview [39]; (ii) known moderate to severe learning disability (*IQ*: < 70); (iii) organic cerebral disease/injury affecting receptive and expressive language comprehension; (iv) non-English speaking to the degree that the participant is unable to answer questions and give written informed consent; and (v) imminent and immediate risk to self or others, operationalised as the presence of active intent or planning to harm oneself or others in the near future (e.g. next month). These criteria would be determined based on self-report and report by the referrer.

Those with experiences of psychosis or bipolar disorder will be excluded since such individuals would typically be referred to secondary care or early intervention services rather than being treated within a university counselling service context. The intervention would require further adaptation for working with those with a learning disability or with communication difficulties; hence, these individuals will also be excluded. No assessment of IQ will be undertaken by the research team; instead, this will be identified based on self-report or report from the referrer. Whilst the focus of the intervention is students struggling with suicidal thoughts or behaviour, we chose to exclude those in acute crisis where there is imminent risk to self. This decision was based on the following: (i) acute crisis management altering the nature and form of the intervention and (ii) the safety of the BMAC in this context still requiring further evaluation. It has the limitation of excluding those individuals presenting with higher risk, which may narrow the sample, but was judged necessary from a safety perspective. Where individuals are excluded because of immediate risk to self or others, with the person’s consent, the researcher will aim to re-contact them and the referrer in approximately 1-month time (or a time period agreed in collaboration with the individual) to determine if risk has subsided to a point where they are eligible.

Where imminent risk is identified, the project team will focus on risk management and ensuring the safety of that individual (regardless of whether that risk is identified before or after obtaining consent). Steps taken will be guided by the project risk management protocol and would typically including sharing of information about the risk with relevant parties (e.g. referrer) and signposting to appropriate services (e.g. crisis and home treatment team). Researchers may support individuals in attending a local hospital emergency department or to wait for an ambulance indicated.

### Recruitment and consent

The research team will recruit participants through HEI counselling services and NHS specialist student mental health services within North-West England. Given the study is a feasibility trial, we decided to focus on a specific geographical area for this initial trial. The northwest of England has a high number of HEIs within a small geographical area and also has elevated rates of suicide compared to the national average and so is a good location for the trial [40].

Service staff will discuss the study with potentially eligible students and, if they consent, will refer them into the trial. Posters placed in counselling services will also advertise the study, and students can self-refer by contacting the research team directly. The team will first contact referrals and self-referrals to ensure eligibility. Eligibility will be assessed via self-report and confirming with the counselling service/referrer. The researcher would then arrange an initial appointment, where they will take consent and complete the baseline assessments. Informed consent will be taken by a researcher using a signed form when the meeting is in-person or verbally where the meeting is remote (e.g. phone or video call). Verbal consent will be guided by a consent script and will be audio recorded via an encrypted device. We opted to use verbal consent for remote meetings as opposed to other methods (e.g. participants mailing back consent forms) as the most immediate and least burdensome approach for participants.

We will encourage recruitment by developing good relationships with referring sites, with whom we will remain in regular, close contact. Having a flexible referral process (e.g. option of clinician and self-referrals) will also help support recruitment.

### Interventions

#### Risk assessment and signposting

Participants in both arms of the trial will be offered up to two sessions involving comprehensive assessment of risk and signposting to further sources of support. Given the anticipated variability in what different counselling services may provide as usual treatment, this will be offered to ensure participants experience the appropriate management of risk. These sessions will include the generation of a collaborative plan with students concerning where they could access further help, advice or support, and what steps they could take if they experience any escalation in their risk. Sessions will last approximately 60 min and will typically be scheduled a week apart (if more than one session).

#### BMAC intervention

Those in the intervention arm will receive the BMAC intervention immediately after the completion of the risk assessment and signposting session(s). The BMAC intervention and the risk assessment and signposting session(s) will be delivered by the same therapist. The initial risk assessment and signposting sessions therefore have the additional benefit of allowing the development of rapport and a positive therapeutic relationship prior to the start of the BMAC intervention. The BMAC intervention will involve up to 6-h-long sessions. All sessions will be completed within an 8-week therapy window.

The theory behind the BMAC is described above, but in brief, the focus will be on strengthening and sensitising networks of positive memories and associated emotions and cognitions, through the use of guided imaginal exposure. Sessions will involve socialisation to the BMAC technique (aided by examples and metaphors), in-session practice and rehearsal, planning for practice outside of sessions, and problem-solving difficulties. Practice of the technique itself will involve identification of a suitable positive memory (avoiding memories associated with more complex or mixed feelings such as loss), undergoing a period of relaxation, before using guided imagery and reimagining of the past event, with a focus on connecting with and reliving the sensory, cognitive, social, and emotional experience of the event.

Practice of the technique between sessions will be encouraged, with prompt sheets and audio recordings to help support this practice. Towards the end of the intervention, a therapy blueprint will be collaboratively developed between the clinician and participant, summarising what was covered in the sessions and highlighting important insights and reflections. A single booster session will be arranged for the 8 weeks following the end of the therapy window, to help further consolidate learning.

#### Therapist and setting

Sessions can take place in-person (e.g. service therapy room, participant’s home, university room) or remotely (phone or video call), allowing flexibility according to patient preference. The BMAC intervention will be guided by a manual developed by the study team based on prior research [34, 41]. A copy of the manual is available upon request at the discretion of the chief investigators. The therapist will be an NHS band 6 professionally qualified and accredited clinician (e.g. nurse, social worker, occupational therapist). This banding was selected to mirror the workforce that is typically available within student mental health and counselling services. Training will be provided in the form of group and one-to-one sessions and workshops, covering the principles and practice of the intervention, using role-plays to aid skill development. Fidelity to the BMAC intervention will be monitored via a sessional checklist completed by the therapist, alongside recordings of sessions, and clinical supervision. The therapist will receive weekly to fortnightly supervision with a qualified clinical psychologist. Whilst the same therapist will deliver both interventions (BMAC; risk assessment & signposting), the two are distinct and clearly specified in the manual to minimise contamination risk. Supervision and review of the sessional checklist will help further minimise contamination. This section is Template for Intervention Description and Replication (TIDieR) compliant [42].

#### Concomitant interventions

Participants will be free to access any other treatments that may be available to them through NHS, university, or other services (e.g. 3rd sector). These interventions are likely to be highly variable across sites and individuals and may include case management, monitoring, and signposting by a counsellor, nurse, or social worker. It may also include medication as prescribed by a GP or psychiatrist. Some students may receive another talking therapy or counselling from a psychologist or psychological therapist. Self-reported receipt of concomitant interventions will be recorded.

### Outcomes

#### Primary outcomes

The primary outcome relates to the feasibility. Data relating to participant flow, referral numbers, consents, attendance, and withdrawals will be collected. The study feasibility criteria are listed in Table 1. All green outcomes would support progression to a larger trial. One or more amber outcomes would indicate modifications to the trial protocol, assessments, or interventions may be required before starting a definitive trial, which could be informed by the qualitative work. One or more red outcome indicates that the trial is unlikely to progress or that substantial modifications may be required before this can happen.

**Table 1:** Feasibility progression criteria with traffic light indicators

Outcome	Criterion	Green	Amber	Red
Recruitment	Ability to randomise 66 participants in an 11-month recruitment window	≥ 80%	60–79%	< 60%
Adherence	Percentage of participants receiving the minimum dose of therapy (≥ 2 sessions) within 8-week treatment window^a^	≥ 80%	60–79%	< 60%
Retention	Percentage of participants completing the 24-week assessment as potential primary outcome timepoint	≥ 80%	60–79%	< 60%
Outcome suitability	Informed by qualitative workstream plus percentage of participants completing the Beck Scale for Suicidal Ideation at all timepoints	≥ 80%	60–79%	< 60%
Safety	Monitoring and review of research-related serious adverse events (SAEs). The Trial Steering Committee (TSC) will oversee SAEs across treatment arms. We will consider discontinuation of the trial if the intervention or procedures are deemed to elevate risk			

^a^Therapy dose does not include the two risk assessment and signposting sessions offered to all participants or the booster session

In addition to quantitative feasibility data, information relating to the acceptability of the intervention and the trial procedures more broadly will be obtained through the qualitative component of the trial. Whilst the progression criterion relating to adherence concerns the BMAC intervention, we will also separately review adherence to the risk assessment and signposting sessions. This information will help to determine the feasibility of this control intervention.

#### Secondary outcomes

Data will also be collected on relevant clinical and mechanistic outcomes. Whilst the trial is not designed to allow for efficacy testing, these data will allow for a preliminary estimation of treatment effects and calculation of relevant statistical properties of potential outcome measures (e.g. standard deviation) that will help to inform future power calculations. We will also consider the suitability of different measures as primary outcomes for a future efficacy trial based on missing data, statistical properties of the scales, and qualitative feedback from participants. The proposed primary outcomes for a future efficacy trial the Beck Scale for Suicidal Ideation (BSS; [43]). Suicide attempt can be problematic as an outcome given the low base rate of the event [44]. Suicidal ideation is a suitable alternative outcome, given that it is a predictor of suicidal behaviour [45, 46], and many theoretical models identify the presence of ideation as a necessary, if not sufficient, condition for suicidal behaviour to occur [47]. Ideation is also often an indicator of broader psychological difficulties and need [15, 48].

In addition to the measures listed below, sociodemographic information (e.g. age, gender, ethnicity, international student status, diagnosis, inpatient admissions, schooling, and living arrangements) will be recorded. We will calculate index of multiple deprivation scores [49] for each participant based on their term and holiday time postcode, given the link between self-harm and socio-economic deprivation [50]. Given the elevated risks of suicide and self-harm amongst LGBTQ + students, sexual orientation and non-cisgender will also be monitored [51, 52]. Table 2 outlines the schedule for when different assessments will be administered.

**Table 2:** Overview of assessment schedule

Assessment	Baseline	8 weeks	16 weeks	24 weeks
Demographics	x	x	x	x
MINI	x	-	-	-
BSS	x	x	x	x
BHS	x	x	x	x
Self-harm	x	x	x	x
PHQ9	x	x	x	x
GAD7	x	x	x	x
PSS	x	x	x	x
SDES	x	x	x	x
PANAS	x	x	x	x
PCISS	x	x	x	x

*MINI* Mini-International Neuropsychiatric Interview, *BSS* Beck Scale for Suicidal Ideation, *BHS* Beck Hopelessness Scale, *PHQ9* Patient Health Questionnaire, *GAD7* Generalized Anxiety Disorder Scale, *PSS* Perceived Stress Scale, *SDES* Short Defeat & Entrapment Scale, *PANAS* Positive and Negative Affect Scale, *PCISS* Perceived Control of Internal States Scale

#### Suicidal ideation

The BSS is a 19-item self-report questionnaire that assesses suicidal ideation (including intent and planning) over the preceding week. The scale is widely used, including in trials of suicide prevention interventions where it has been employed as a primary outcome (e.g. [53]). The BSS has good reliability and validity [54], and the scale has measurement invariance over time, making it suitable as a longitudinal follow-up [55].

#### Self-harm and suicidal behaviour

Self-harm will be assessed using the items ‘At any time in your life/since the last assessment, have you deliberately harmed or injured yourself or attempted suicide?’ and ‘How many times have you deliberately harmed or injured yourself or attempted suicide in your life/since the last assessment?’ from the Linehan Suicide-Attempt Self-Injury Interview [56]. Additional items from the suicide attempt and non-suicidal self-injury modules of the Self-injurious Thoughts and Behaviours Interview [57] were used to obtained further information regarding self-harm and to distinguish between suicidal and non-suicidal self-injury.

#### Hopelessness

The Beck Hopeless Scale (BHS; [58]) is a 20-item questionnaire assessing feels of hopelessness within the last week. Studies have supported the reliability and validity of the BHS [54, 58, 59]. Evidence regarding the optimal factor structure for the BHS varies, but a recent study supports the use of a three-factor model over alternatives [60]. This trial therefore adopts the total score and these three subscales which capture feelings about the future, loss of motivation, and future expectations.

#### Depression, anxiety, and stress

The Patient Health Questionnaire (PHQ9; [61]) and the Generalized Anxiety Disorder scale (GAD7; [62]) will be used to measure depressive and anxiety symptoms, occurring within the last week or 2 weeks, respectively. Both measures demonstrate good reliability and validity [61–63]. The perceived stress scale (PSS; [64, 65]) will also be used as a measure of stress over the preceding month. This scale also has been shown to have good reliability and validity, with the 10-item version that is being used in this study having supervisor properties [65]. Whilst there is debate around the optimal factor structure for this scale, a single scale total has been recently recommended for university students [66].

#### Defeat and entrapment

The Short Defeat and Entrapment Scale (SDES; [67]) provides a brief, 8-item assessment of feelings of being defeated or trapped, which has been adapted from the longer defeat and entrapment scales [68]. The SDES has had its factor structure, reliability, and validity, including associations with related constructs such as hopelessness and its ability to distinguish clinical and non-clinical groups [67].

#### Perceived control and affect

We hypothesise that the BMAC will act on suicidal thoughts and behaviours by increasing participants’ access to and perceived control over desirable thoughts and emotions. We will therefore employ the Positive and Negative Affect Schedule (PANAS; [69]) to assess positive and negative emotional states, and the Perceived Control of Internal States Scale (PCISS; [70]), which measures perceived control over thoughts, emotions, and bodily sensations.

#### Psychiatric difficulties

Current and past psychiatric difficulties will be assessed as the baseline assessment, using the Mini-International Neuropsychiatric Interview [39], to describe the diagnostic composition of the sample. Researchers will receive training in the administration of this interview from an experienced clinician. This interview will also be used to confirm the absence of bipolar disorder or psychotic episodes, as noted above. The interview modules relating to suicidality will not be used given this is assessed by several other measures. The modules relating to eating disorders and antisocial personality disorder will also not used in order to minimise participant burden.

### Data collection

Assessment sessions can take place in-person (at participants’ home, university campus, or health service) or remotely (e.g. phone or video call) depending on preference and practicality, and participants will be able to undertake some assessments in-person and others remotely. This allows for greater flexibility in arranging appointments, recommended by patient and public involvement consultants, and helps protect the study from any future social distancing linked to the COVID-19 pandemic. Appointments will be arranged with the researcher. Follow-ups can occur within a 3-week window, up to 1 week before and up to 2 weeks after the follow-up date, as nearer to the follow-up date as possible. Researchers will send participant reminders by email, phone, or text prior to assessment meetings. Participants will be able to choose to withdraw from therapy but remain in the trial (e.g. continue to complete assessments) or to withdraw from the trial as a whole, at any time. In both cases, reasons for this would be sought.

Where possible, assessments are completed within a single meeting, but breaks can be taken, and an assessment may be completed over several sessions where a participant requests this (e.g. where they do not have time to complete all measures in one session). In the latter case, baseline assessments still should be completed within 2 weeks of having started and follow-up assessments within 1 week of having started. Data is collected via a paper case report form (CRF), which is completed by the researcher. Copies of the CRF (excluding copyrighted material) are available on reasonable request from the study team. Researchers will receive training and regular research supervision to support their administration of assessments.

### Qualitative evaluation

An embedded qualitative evaluation will be used to provide further data concerning the feasibility and acceptability of the trial and BMAC intervention. This evaluation will include investigation of potential barriers and facilitators to uptake, perceptions and experiences of both delivering and receiving the intervention, and ways of optimising acceptability and feasibility for a future trial. Potential mechanisms of change within the intervention will also be investigated. A subsample of up to twenty participants from the wider trial will be sought to take part in individual qualitative interviews. Participants will be asked when providing consent if they also wish to be contacted about taking part in this qualitative interview. A subsample of those that agree to this will then be invited to take part in the interview following the 8-week follow-up point. Participants will be sampled from both arms of the trial, allowing for data concerning the experience of the BMAC intervention, but also the experience of being allocated to the control arm, to be captured. Interviews will be undertaken with a non-blinded researcher guided by an interview schedule, which has been developed with support from the trial advisory group. Trial research staff and therapist(s) will also be invited to qualitative interviews, investigating their experiences and insights of undertaking the trial. The interviews will be supplemented with data from reflective logs that will be kept by trial therapists and research assistants, which will provide data on feasibility challenges as they arise.

### Trial oversight

The Trial Management Group (comprising the coinvestigators, the trial statistician, and trial manager) will meet monthly to oversee the running of the project. Weekly operational meetings with research staff working on the project will support the day-to-day running of the trial. A TSC will meet at least twice annually to provide independent guidance and oversight, consisting of experts in student mental health, suicide, self-harm, and psychological interventions, people with lived experience, statisticians, and clinicians. The TSC includes seven independent members and one non-independent member. A TSC charter has been drafted that outlines the remit and responsibilities of the TSC.

### Patient and public involvement

Patient and public involvement (PPI) informed the trial development. This helped guide various aspects of the trial, including the choice of outcomes, and the format of assessment and therapy meetings. An advisory group consisting of current or former university students with lived experience of suicidal thoughts or behaviours will also meet four times a year for the life of the project to provide further advice, guidance, and feedback related to the ongoing trial.

### Safety monitoring and reporting

Adverse events (AEs) will be monitored throughout the trial. To support this, all follow-up assessments include two questions to routinely monitor for adverse events: “Since the last assessment, have you experienced any life-threatening events or near death experiences?” and “Since the last assessment, has anything happened that has caused you persistent or significant disability or incapacity?” These open questions are worded broadly to increase the chance of identifying any experiences that may constitute AEs. Given the risk and scale of the trial, there will not be a separate data monitoring and ethics committee (DMEC). Instead, the TSC will fulfil the functions of the DMEC, including the monitoring of serious adverse events (SAEs) and determination of whether these constitute reactions to trial procedures or therapy (i.e. serious adverse reactions). Given this role, extraordinary meetings of the TSC or a subgroup of the TSC tasked with data monitoring may be convened by the TSC chair as needed. Where adverse reactions are identified, the intervention and/or trial may be discontinued for particular participants or as a whole. This will be determined based on discussions between the project team, TSC, project sponsor, and ethics committee.

### Sample size

A sample size of 66 participants will enable investigation of the main research questions regarding feasibility and acceptability. It will enable estimation of the retention rate to within approx. + / − 11% with 95% confidence, assuming the retention rate is no less than 70%, and sufficient data to estimate the SD of suicidal ideation [71, 72]. Sample size for the qualitative evaluation will be determined by data sufficiency so that adequate data will be collected to answer the research questions [73].

### Data management

A trial data management and monitoring plan will be developed. An electronic participant tracking log will be used to keep track of participant progression within the trial. Study assessment data will initially be collected on paper CRF, which will be scanned to produce electronic copies. Data will be entered into an electronic spreadsheet as soon as possible. Planned visual checks of a 10% subset of electronic data against paper records will be undertaken. Clinical data including randomisation information will be stored in electronic files separately to other study data to maintain blinding and only combined upon trial completion. Qualitative interview recordings will be transcribed, de-identified, and stored electronically, with audio recordings deleted following transcription. Participant identifiable information will be stored separately to other study data, with a confidential ID code used to the link the two. Electronic study data will be stored on a secure, password-protected NHS computer drive that can only be accessed by the study team. Files will also be password-protected. Hard documents will be stored within locked filing cabinets within a secure NHS building. Upon completion of data collection, outcome data, relevant clinical data (e.g. attendance information), and treatment allocation data will be merged into a single anonymised dataset. Anonymised electronic copies of the dataset will then be shared with the trial statistician for analysis.

### Data analysis

Examination of the feasibility outcome data will take place after the end of the last follow-up assessment. This will include reporting data in line with the updated Consolidated Standards of Reporting Trials 2010 statement for randomised pilot and feasibility trials, summarising recruitment and attrition rates, willingness to consent, attrition, and missing data [74]. Outcome data, including potential mediators, will be summarised to inform the future definitive trial, including presenting the mean and standard deviation of outcome measures (both overall and by treatment group) and the difference between intervention group means (with 95% confidence interval). Analyses will adopt an intent-to-treat approach. No imputation will be used in the analysis, but we will report percentages of missing data for each follow-up time-point and each questionnaire (including item missing data where this appears relevant). We will further consider the potential effect size in relation to published minimally (clinically) important differences by extending confidence interval estimation to include 70%, 75%, 80%, 85%, and 90% confidence intervals, following the recommendation of Lee and colleagues [75], using a regression-based approach, adjusting for the baseline measure of the respective outcome. Bootstrapping will be used to provide bias-corrected percentile intervals given the likely non-normality of outcome measures. Further details of the analysis will be included in a statistical analysis plan which will be developed, with input from the Trial Steering Committee, and approved prior to any unblinded review or analysis of outcome data.

For the qualitative data, interviews and field notes will be analysed using reflexive thematic analysis [76, 77] to identify patterns of themes across the data corpus. Thematic analysis will utilise Braun and Clark’s recommended process starting with intensive reading of transcripts [76]. Qualitative analysis will proceed simultaneously with data collection to allow emerging information to be incorporated and explored in subsequent interviews. The research associate will lead on initial coding and generation of themes, in consultation with the wider team and stakeholder consultation group. This will allow for multiple perspectives on the data during analysis. Coding will be conducted systematically and iteratively using appropriate coding software (NVIVO; [78]). Quotes will be anonymised to avoid individuals being identifiable.

### Ethics and auditing

The project has been approved by an NHS research ethics committee (London — Bromley REC; ID: 305,348). Any modifications to the study protocol affecting the conduct of the study or the experience of participants would be reviewed by the TSC and (depending on the nature of the modification) the project sponsor (Greater Manchester Mental Health NHS Foundation Trust) and funder (National Institute of Health and Care Research). An ethics amendment would be sought as required before implementing any such modifications.

There are no planned audits from external organisations. However, data from the project may be audited at any point by relevant agencies from the project sponsor or partners. We will make this clear to all participants before they agree to take part in the study. The trial management team will monitor study protocol adherence. The TSC and sponsor will also be able to request audits.

### Dissemination

The trial results will be disseminated through multiple diverse routes. These include peer-review publications and presentations at academic conferences but also press releases and lay summaries (shared on websites and via relevant organisations). An infographic will be developed with the project advisory group and a graphic designer ,and shared online and through participating organisations. A full report will be submitted to the project funder. The trial management team will agree authorship on publications in advance of submission. Decisions about authorship will follow recognised international guidelines (e.g. https://www.apa.org/research/responsible/publication), and disputes concerning authorship would be resolved through discussion with the TSC.

## Discussion

Suicide amongst university students is a growing concern. Brief targeted psychological interventions, which could be readily embedded within university counselling or student mental health services, have great potential in helping to support those students struggling with suicidal thoughts and feelings and to reduce suicide risk. Providing effective psychological intervention at what is a key time for many people may also help generate longer-term resilience. The BMAC intervention has so far shown promise in small-scale pilot studies and proof-of-concept experiments, but requires evaluation on a larger scale. MISST represents the next step in evaluating the BMAC intervention for university students struggling with suicidal ideation or behaviour. MISST follows best practice guidelines for feasibility RCTs, including robust procedures adopted for randomisation and blinding, the use of PPI, and inclusion of qualitative evaluation. If MISST meets its progression criteria and demonstrates promise, then the next step would be to seek funding for a large-scale efficacy trial in order to determine the clinical and cost effectiveness of the approach.

### Trial status

This paper presents protocol version 2 (13/02/3022). Trial recruitment started in February 2022 and is due to finish in January 2023.

## Supplementary Information

Below is the link to the electronic supplementary material.

Additional file 1: Table 1: SPIRT Checklist.

## Data Availability

An anonymised version of the final trial dataset will be made available to other researchers following publication of the study, at the discretion of the authors.
